# Contradictory Effect of Notch1 and Notch2 on Phosphatase and Tensin Homolog and its Influence on Glioblastoma Angiogenesis

**DOI:** 10.31661/gmj.v10i0.2091

**Published:** 2021-09-28

**Authors:** Mostafa Shabani, Hamid Taghvaei Javanshir, Ahmad Bereimipour, Amin Ebrahimi Sadrabadi, Arsalan Jalili, Karim Nayernia

**Affiliations:** ^1^Medical Genomics Research Center, Tehran Medical Sciences Islamic Azad University, Tehran, Iran; ^2^Department of Stem Cells and Developmental Biology, Cell Science Research Center, Royan Institute for Stem Cell Biology and Technology, ACECR, Tehran, Iran; ^3^Young Researchers and Elite Club, Tehran Medical Sciences Islamic Azad University, Tehran, Iran; ^4^International Center for Personalized Medicine, Düsseldorf, Germany

**Keywords:** Glioblastoma, Angiogenesis, Notch1, Notch2

## Abstract

Many genes induce angiogenesis in tumors, and among them, Notch family genes have received particular attention due to their extensive network of connections with other genes active in this function. Suppression of angiogenic signaling has been studied in various cancers, confirming Notch's fundamental and extensive role. According to studies, four Notch genes work independently with many genes such as vascular endothelial growth factor, phosphatase and tensin homolog, Phosphoinositide 3-kinase/Akt, and matrix metalloproteinases, and so many other genes, as well as proteins (such as hypoxia-inducible factor-1 alpha) significantly affect tumor angiogenesis. Notch1 regular activity in a healthy person causes angiogenesis in body tissues, controlled by normal Notch2 activity. However, in many cases of glioblastoma, whether on patients or tumor xenografts or in vivo models, a mutation in one of these two essential genes or at least one of the genes and proteins that affected by them can cause better angiogenesis in hypoxic conditions and lead to become an invasive tumor. In this review, we examined the contrasting activity of Notch1 and Notch2 and the signaling cascade that each generates in the angiogenesis of glioblastoma, the most invasive cancer of the central nervous system.

## Introduction

Glioma is the most malignant type of primary brain tumor that is highly resistant to chemotherapy and other medications. According to the World Health Organization (WHO) classification, glioma with rapid cell proliferation and high resistance to treatment and optimized angiogenesis consist of four histopathological degrees (I-IV), including pilocytic astrocytomas, diffuse astrocytomas, anaplastic astrocytomas, and type IV glioma astrocytoma or glioblastoma multiform (GBM) [1]. Astrocytes are the most abundant type of glial cells in the brain that are multifunctional cells with different central nervous system (CNS) roles and can cause several diseases. They also can control nerve synapses, regulate homeostasis, supply energy to neurons, and recycle neurotransmitters [2,3,4]. GBM accounts for about 70% of all gliomas [5]. The current standard of care for patients with GBM includes surgical intervention with maximum possible tumor resection, followed by concomitant radiotherapy (RT) and chemotherapy with temozolomide (TMZ) and then the adjunctive drug TMZ, which is an oral alkylating agent; due to its small size, it is easily absorbed in the intestine and passes through the blood-brain barrier [6,7]. Despite these invasive treatments, patients' maximum survival time is estimated at 14.6 months, with a mortality rate of nearly a hundred percent [8,9]. Tumor angiogenesis is regulated by a complex network of signaling pathways, including vascular endothelial growth factor (VEGF), epidermal growth factor receptor (EGFR), peritumoral brain edema (PTBE), P53, and Notch. Although not all of the mechanisms involved are known, the Notch signaling pathway is critical in tumor angiogenesis [1]. Notch signaling plays an important role in regulating many stem cell processes such as proliferation, stem cell maintenance, differentiation during embryonic and adult development, homeostasis of adult regenerative organs, and CNS development [10,11]. In mammals, Notch consists of four hetero-oligomer single-pass types I transmembrane receptors (Notch 1-4) and five ligands from the Delta-Serrate-Lag family, including Jagged1 (JAG1) and JAG2, delta-like 1 (DLL1), DLL3, and DLL4 belong to the Serrate family of ligands. Notch receptors are heterodimers with extra- and intracellular functional domains that mediate the target gene's transcription [1]. According to reviewed information and the Notch signaling pathway's crucial and contradictory function in inhibiting or inducing angiogenesis, this study aims to determine Notch1 and Notch2 in the signaling current that each creates and finally finds a suitable solution to inhibit angiogenesis in GBM or suggest reliable treatment.

## Notch Signaling Expression Pattern in GBM

Notch signaling pathway is disrupted in three-quarters of human GBM, and its growth is suppressed explicitly by inhibiting a receptor in the Notch family [12,13,14]. Notch1, Notch4, DLL1, DLL2, JAG1, Centromere-binding protein-1 (CBF1), Hairy/enhancer-of-split related with YRPW motif protein-1 (HEY1), HEY2, and hairy and enhancer of split-1 (HES1) mRNA and protein levels are higher in brain tumor cells than in normal brain tissue, and also with increased VEGF and Phosphorylated protein kinase, strain AK, Thymoma (pAKT) expressions, and decreased Phosphatase and tensin homolog (PTEN) levels [15,16,17]. For example, higher expression of Achaete-Scute Family BHLH Transcription Factor-1 (ASCL1), DLL1, Notch1, Notch3, Notch4, and Hey1 is associated with high-grade glioma and a worse prognosis [18,19]. As a result, the more active Notch signaling, the more differentiated and aggressive the tumor phenotype. Some research also suggests that Notch activation creates a specific cellular fate, especially distinguishing certain glia types, such as radial glia and astrocytes [20,21].

1.1. Notch1 Signaling Overview

According to studies, among the four Notch genes active in GBM angiogenesis, Notch1 plays the most crucial role. It works in low oxygen conditions concentration with the participation of Hypoxia Inducible Factor (HIF-1α). Suppression of Notch1 impairs the proliferation and survival of glioma cell lines as well as human gliomas. Notch1 expression is higher in patients with a chance of survival of more than one year than in less than one year of age. However, Notch1 overexpression is less associated with overall survival (OS), suggesting a controversial role for Notch1 in GBM [22,23]. Microglia/macrophages enhance glioma growth by secreting proteolytic enzymes and several angiogenic factors such as matrix metalloproteinases (MMPs), VEGF, and affecting nuclear factor kappa B (NF-κB) [24,25,26]. NF-κB and MMPs are involved in tumor cell invasion and tumor angiogenesis, inactivated by Notch1 inhibition [27]. Besides, the limiting effect of Notch1 on PTEN signaling has been observed [28].

1.2. Notch2 Signaling Overview

Notch2 is known to be a significant prognostic marker in glioma independent of other mutation patterns [29]. The level of Notch2 expression in GBM is associated with stem genes (nestin and SRY-Box Transcription Factor-2), fate genes (expected outcome of normal development), astrocytes (vimentin and Glial Fibrillary Acidic Protein), and anti-apoptotic proteins (BCL6 and BCL-W) but is inversely related to Oligodendrocyte Transcription Factor-2 (Olig2), C-type natriuretic peptide (CNP), and PLP1 (oligodendrocyte fate) and proapoptotic proteins (apoptosis promoters) such as Bcl-2-associated X protein and Bcl-2-associated transcription factor 1 [30,31]. Positive regulation of Notch2 expression effectively suppresses cell growth and invasion and induces apoptosis [1]. In a study of the malignant mesothelioma, an invasive tumor of the pleura, pericardium, and peritoneum, Notch2 negatively regulated PI3k-Akt and found that Notch2 could activate PTEN, both of which inhibition of angiogenesis plays a pivotal role [28]. Another study showed that negative Notch2 regulation induced by siRNA in gastric cancer cells increases the invasive function of tumor cells, enhances the expression and activity of MMP9, and increases the phosphorylation of the PI3K pathway, as with growing p-Akt was shown [32].

1.3. Notch3 Signaling Overview

Notch3 is a prognostic factor expressed in the CNS, vascular smooth muscle, and some hematopoietic cells; and enhances cell proliferation, migration, and invasion, which inactivates cell apoptosis [1و^33^]. Activation of Notch3 causes invasive glioma formation in the optic nerve but has no confirmed effect on GBM. It has also been observed that Notch3 increases expression in hypoxic conditions and contributes to significant angiogenesis [34,35,36,37,38,39]. Some studies suggest Notch3 has an impact on EGFR gene expression, which could potentiate PI3K function. Also, research shows that Notch3 plays an essential role in fibroblast-dependent angiogenesis [40,41].

1.4. Notch4 Signaling Overview

High expression of Notch1 indicates higher differentiation, while increased expression of Notch4 may indicate a lower degree of differentiation and possibly a tumor with more aggressive function [30]. Not much is known about the cooperation of Notch4 with other genes affecting glioma, and more studies are needed. Information obtained by Uyttendaele et al. [42] shows that among the components of the DLL4-Notch pathway, DLL4 and Notch4 are expressed explicitly in tumor endothelial cells. This important association and specific morphology showed that DLL4-Notch4 signaling in endothelial cells plays a vital role in GBM angiogenesis. It has been suggested that Notch1 and Notch4 may have similar functions in angiogenesis, regulating, and acting on them in different combinations in different cell types [42]. In summary, the functional role of Notch1 and Notch2 compared to Notch3 and Notch4 in influencing GBM is better known, scientifically proven, and has a meaningful place in angiogenesis research and the challenging effect of these two genes. Also, their signaling pathway has been studied in various cancers. Numerous articles have examined the promoting or limiting impact of Notch1 and Notch2 on cell lines, tumor xenografts, animal models, and human tumor specimens.

## 2. Notch Signaling and Angiogenesis

Blood vessel formation is a dynamic and complicated process that plays a crucial role in health state and disease vulnerability. Delicate balance-dependent angiogenesis is regulated between anti-angiogenic and pro-angiogenic molecules and angiogenesis in tumors. Angiogenesis occurs when pro-angiogenic stimuli are more potent [43,44]. Among the many signaling pathways that affect angiogenesis, the Notch signaling pathway is a ligand-receptor cascade that plays a vital role in guiding cellular fate and vascular development and inducing tumor angiogenesis [45,46]. Two key ligands mediate Notch paracrine receptors in glioma stem cells (GSCs), DLL4, and JAG1, expressed in epithelial cells (ECs) [47].

2.1. DLL4

DLL4 is a Notch1 ligand that plays a vital role in vascular development and is present in active angiogenesis sites. Predictors of tumor progression and survival are independent of age, sex, WHO grade, PTBE, and expression levels of Ki-67, MGMT, and p53 [48,49,50,51,52]. Li, Z. et al. showed that in different ECs classes, hypoxic conditions lead to induction of DLL4 by HIF-1α [50]. DLL4 levels in tumors and vascular tissue are considered as predictive markers [53]. DLL4-Notch signaling pathway interacts with several molecules and other signaling pathways, including PI3k, EGFR, and MMP9, all related to tumor invasion, proliferation, and metastasis. Notch1-dependent activity in the PI3k-Akt pathway via DLL4 leads to cell migration and invasive cancer [17,54,55]. According to previous observations, overexpression of DLL4 in glioma connective tissue reduces vascular density, improves vascular collapse, reduces intra-tumor hypoxia and necrosis, and ultimately prevents tumor growth. In contrast, inactivating DLL4 causes unproductive angiogenesis (production of a dysfunctional vessels network) with necrosis and hypoxia. DLL4-Notch signaling activity in tumors enhances blood vessels' better perfusion (productive vessels), stimulating tumor growth despite the reduced vascular density and improving function within a tumor [56,57]. Besides, the Notch pathway also regulates tumor cell differentiation into ECs in several ways [14,58,59].

2.2. JAG1

The Notch /JAG1 signaling pathway can work directly with other essential pathways such as MMP9 and VEGF to regulate glioma growth and malignancy, which defines patients’ physical condition with glioma [60]. Numerous articles indicate the JAG1 signaling pathway as a modulator of angiogenesis associated with DLL4, with JAG1 somewhat limiting DLL4 function to keep it out of control. In other words, JAG1 and DLL4, as Notch1 ligands, together cause normal angiogenesis, and any dysfunction of either causes inefficient angiogenesis and invasive tumor [61].

2.3. HES1

HES1 is a transcription factor and downstream target in the Notch1 signaling pathway. According to studies, it is located on human arterial ECs, and its significant effect on the regulation and morphologic changes of angiogenesis is confirmed [62]. Tumor growth factor-α (TGF-α) can also regulate HES1 expression independently of Notch1 function and introduce HES1 nuclear import in the presence of ERK1/2 activation. They synergistically promote the growth of glioma cells [63]. One of the critical tasks of HES1, which has been mentioned in various studies, is to cooperate in the development of tumor angiogenesis under the control of Notch1 and inhibit PTEN function [64,65,66,67]. The importance of DLL4 and JAG1 in their complementary function is that the expression of DLL4 in GBM is limited to endothelial cells. It is significantly more common and severe than JAG1; DLL4/Notch angiogenesis's high activity exacerbates GBM. HES1 transcription factor's role as Notch1 operating lever in the control and inhibition of PTEN was investigated in many studies.

##  3. Factors That Affect Notch Performance

Many molecular components are involved in and affect the Notch signaling pathway, the most important of which is hypoxia, affecting Notch1/2 performances through the HIF-1α induction factor. This effect can be inhibitory or inductive. Another important pathway leading to angiogenesis regulated by Notch1/2 and having significant hypoxia activity is PI3k-Akt-MMP9, which is inhibited by the PTEN removed on chromosome 10 [68,69,70,71,72].

3.1. Hypoxia Regulate Angiogenesis in GBM

Tumor angiogenesis is essential for tumor growth and progression, and solid tumors often have increased hypoxia, a potent angiogenesis stimulus. Low oxygen levels may be due to structural abnormalities in the tumor vessels or the tumor size, leading to inadequate oxygen delivery. Changes in gene expression may help the tumor adapt to its hypoxic environment. One of the induced genes is HIF1. HIF1 is a heterodimeric protein belonging to the basic helix–loop–helix family of transcription factors. It regulates the expression of many genes involved in tumor progressions, such as VEGF and Notch [73]. Before the induction of angiogenesis, cells survive in the tumor mass inside and away from blood vessels in nutrient deficiency conditions and inadequate oxygen supply. The association between hypoxic conditions and the Notch pathway in GMB has been reported in several studies [68,69,70,71,72,74,75]. Under hypoxic conditions, the presence of HIF-1α increases the stability of the Notch protein and the physical interaction between the two proteins [68]. Hypoxia is one of the hallmarks of GBM, and Notch signaling works best with hypoxia. Five hypoxia markers (HIF-1α/PGK1/VEGF/CA9/OPN) have been identified as the best predictors of Notch1, DLL1, HES1, HES6, HEY1, and HEY2 induction. Also, under hypoxia, GSCs express several Notch-related genes (Notch1, Notch3, DLL1, JAG1, JAG2, HES1, HEY1, HEY2) and hypoxia-related genes (HIF-1a, VEGF, LOX, and HIG2) increases [69,70,71]. HIF-1α induces Notch pathway activity and makes GSCs more sensitive to maintenance in hypoxic conditions. Hypoxic conditions by activating Notch signaling lead glioblastoma cells to increase colony formation, increase cancer stem cell markers' expression, increase neurosphere production, and malignancy. Various mechanisms are involved in the proper formation of blood vessels observed in these tumors. The germination of capillaries caused by existing blood vessels through endothelial proliferation depends on hypoxia at the tumor center [72]. Hypoxia induction factor, which enhances transcription of VEGF, is activated in GBM. Hypoxic tumor cells, especially cell around the necrotic nucleus, release vascular growth factors such as VEGF, which stimulates the formation of new blood vessels from existing normal endothelial cells [72]. The researchers also explained that Notch activation due to hypoxia could be reversed through targeted Notch therapy. Contrary to the role of HIF-1α in Notch signaling, the reaction of HIF-2α and Notch intracellular domain suppresses Notch signaling. HIF-1α and HIF-2α bind competitively to the Notch intracellular domain and dynamically regulate Notch signaling activation in GSCs depending on different oxygen stresses (concentration changes), and improved treatment opportunities provide oxygen for various strains [74,75].

3.2. Over Expression of PI3k-Akt Signaling Cause Angiogenesis 

One of the Notch1 angiogenesis mechanisms is the induction of PI3k-Akt pathway activity via DLL4. PI3k/Akt signaling regulates angiogenesis by affecting expressions of VEGF, HIF-1α, and MMP9 [57,76,77,78,79]. Activation of PI3k by Notch1 also stimulates the signaling pathways of MMP9, β-catenin, and NF-κB, which increases the migratory, invasiveness, and angiogenic properties of glioma cells [17,77,78]. Inhibition of the PI3K pathway not only limits tumor cell growth but also inhibits tumor angiogenesis. Interestingly, the PI3K pathway plays an influential role in regulating VEGF and VEGFR [80]. Fibroblast growth factor receptor (FGFR) modulates several tumor cell processes, including FGF-mediated migration and proliferation. FGFR plays a substantial role in the survival and angiogenesis of glioblastoma cells via the PI3k/AKT/mTOR signaling pathway [81].

3.3. MMP9 Effects On Angiogenesis 

MMP9 is a family of related enzymes that destroy the extracellular matrix and are essential factors in facilitating tumor invasion and metastasis [82,83,84]. MMP9 is a downstream target for the PI3k/Akt pathway that is crucial in cell proliferation control [77,78]. Under physiological conditions, MMP9 plays a vital role in tissue repair in connection with various physiological and pathological processes such as morphogenesis, angiogenesis, tissue repair, cirrhosis, osteoarthritis, and metastasis [85,86]. MMP9 is required to maintain normal/healthy tissue structure and epithelial integrity. Abnormal expression and activity of MMP9 have been reported in pathological conditions, especially in various cancers [85,86,87,88,89,90].

3.4. PTEN Controls PI3K/AKT Activation

PTEN is a tumor suppressor that neutralizes the PI3K/Akt/mTOR pathway with its lipid phosphatase function. Mutation and methylation of PTEN have been detected in at least 60% of GBM [91]. PTEN may contribute to gliomagenesis and survival by impairing proliferation, migration, invasion, angiogenesis, stem cell self-renewal, and regulation of other tumor suppressor pathways such as P53, poorly associated with glioblastoma [91,92,93,94,95]. It has been observed that Notch1 and Notch2 have different effects on PI3k-Akt signaling with opposed regulation of PTEN, which was confirmed by protein and mRNA level analysis. PTEN activity can also have a limiting impact on HIF1-α and VEGF. Moreover, thereby inhibiting angiogenesis and tumor survival [28,79]. Specifically, in GBM, PTEN loss leads to the expression of VEGFR2 in tumor cells, which may play a role in resistance to angiogenesis inhibitory therapies. A new study also showed that overexpression of VEGFR2 in tumor cells could induce early GBM resistance to TMZ chemotherapy and anti-angiogenic therapy with bevacizumab [96]. Taken together, it seems that Notch1 and its signaling pathways such as PI3k-Akt and MMP9 play a significant role in tumor angiogenesis with the help of VEGF and HIF1-α factors; admittedly, the presence of hypoxia plays a significant role in exacerbating angiogenesis, and in normoxia, less pro-angiogenic factors will be present. Unlike Notch1, Notch2 has a more substantial role in tumor growth and plays a suppressive role in tumor angiogenesis through PTEN induction and AKT dephosphorylation [28]. Defects in the expression or function of PTEN are also indirectly associated with anti-angiogenic drug resistance [96].

## 4. Influence Inhibition of Notch1 and Notch2 Induced Expression On Angiogenesis

**Figure 1:**
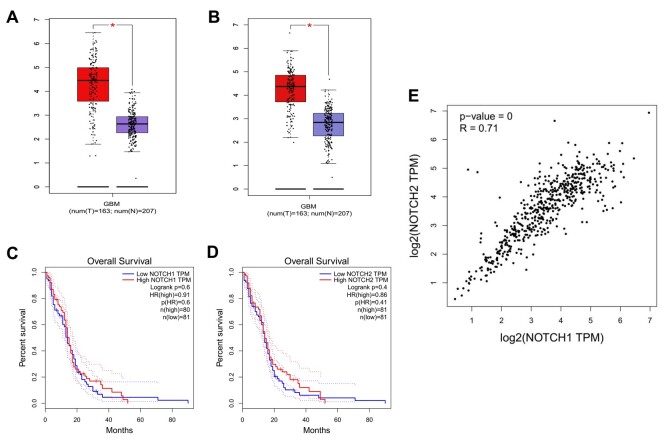
Evaluation of expression, survival, and correlation between Notch1 and Notch2 in GBM.** A and B:** The expression level of Notch1 and Notch2 is shown as a box plot with a significantly higher expression rate in patients with GBM than healthy people.** C and D:** The survival chart between these two genes is about 45 months; the survival rate in patients with high expression of these genes is close to zero.** E: **Correlation between two genes has been shown that with increasing expression of each gene, the other gene has also increased expression. It can be said that these two genes are related to each other. The diagrams were drawn using the GEPIA database.

**Figure 2:**
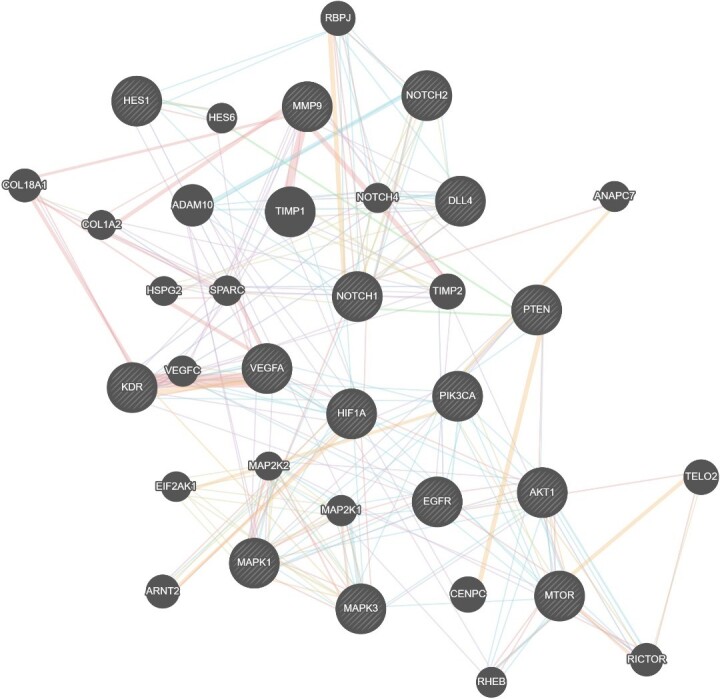
The network drawn using the GeneMANIA database shows the relationship between the upstream and downstream genes Notch1 and Notch2. The circles and the connections between the lines of each gene show the importance of that gene in the network.

Due to Notch1 and Notch2 proteins' contradictory function in GBM angiogenesis, regulating their activity and downstream signaling for therapeutic methods has been investigated in many articles. In a single-gene therapy study and multi-gene combinatorial therapy on EGFR, PI3K, AKT, and PTEN in GBM, Han et al. reported that PTEN was upregulated by adenoviral-mediated PTEN (Ad-PTEN), and PI3K was suppressed by LY294002 (Figure-1, Figure-2) [95,97,98]. The effect of this combination therapy was evaluated on glioma cell lines (U251 and LN229) and tumor xenograft (U251). Although multi-gene combination therapy is far more effective than selective gene therapy, it still cannot completely inhibit glioma growth, and further studies are suggested [95,97,98]. >In another study, researchers used Notch1 siRNA to reduce Notch1 function and increase Plasmid-induced Notch2 expression. Notch1 siRNA transfer to GSCs suppresses the Notch1 gene. Notch1 mRNA level and according to Western blot analysis, Notch1 protein was significantly reduced in this group compared to the control group [99,100].

## Conflict of Interest

The authors declared that they have no conflict of interest.
